# Metabolic syndrome among students attending a historically black college: prevalence and gender differences

**DOI:** 10.1186/1758-5996-5-2

**Published:** 2013-01-12

**Authors:** Avinash M Topè, Phyllis F Rogers

**Affiliations:** 1 Division of Food and Animal Science, College of Agriculture, Food Science and Sustainable Systems, Kentucky State University, Frankfort, KY, 40601, USA

**Keywords:** College students, Lipids, Pre-diabetes, Metabolic syndrome, Young African American adults

## Abstract

**Background:**

There are limited data on the prevalence rate of Metabolic Syndrome (MetS) among college students attending any Historically Black College and University (HBCU), which are mostly attended by young African Americans (AA). We report the prevalence and gender differences in the components of MetS in a sample population from an HBCU campus.

**Methods:**

Three hundred and seventy six (218 females and 158 males) first year college students (average age 19.8 years), attending Kentucky State University, Frankfort with no prior diagnosis of illness participated in the cross sectional study. Anthropometric screenings included measurement of height, weight, waist circumference and body mass index (BMI). The clinical screenings included measurement of blood pressure and determination of fasting lipid and glucose concentrations. The National Cholesterol Education Program’s Adult Treatment Panel III (NCEP ATP III) and International Diabetes Federation (IDF) definitions for MetS were applied. *Statistics*: Analysis of variance (ANOVA) scores on the Means procedure were used to examine differences between genders for all anthropometric, clinical and biochemical parameters. Fisher’s exact chi-square tests were used to analyze the prevalence of MetS criteria per gender, the number of MetS criteria per BMI category and the prevalence of MetS criteria. Significance was set at *p* ≤ 0.05 for all tests.

**Results:**

Prevalence rates for MetS criteria varied depending on the definition used. According to the NCEP ATP definition, 31.4% of the sample population had at least 1 criterion for MetS, while 20.7% had 2 criteria. When IDF definition was applied, 21.3% sample population had 1 criterion and 17.5% had at least two criteria. Prevalence was highest for low levels of high-density lipoprotein cholesterol (37.3%) and elevated fasting glucose (22.1%). On the basis of the NCEP ATP and IDF definitions, overall prevalence of MetS in the total sample was 12%, and 9.3% respectively.

**Conclusions:**

HBCUs offer a unique opportunity to monitor and address the risk factors of MetS in a predominantly young AA population. There is a higher prevalence of MetS in this study population than any other reports on college students.

## Background

Metabolic Syndrome (MetS) is a cluster of interrelated cardio-metabolic risk factors that include insulin resistance, lipid imbalance and hypertension [1-4]. It is considered to be a precursor of coronary heart disease (CHD) and diabetes [5,6]. It is associated with morbidity and all causes of mortality [7,8]. In the United States (US) CHD is the second leading cause of all natural deaths in young adults ages 18–24 [9]. However, it is ‘the leading’ cause of all natural deaths in non-Hispanic AA in this age group [10]. According to the 2003–2006 National Health and Nutrition Examination Survey (NHANES) in the US population, the prevalence of MetS in males and females, ages 20–39 years was 20.3% and 15.5% respectively [7]. Although its prevalence increases with age and Body Mass Index (BMI), the individual risk factors also vary between races and ethnicities. Its overall prevalence is particularly high among AA and Hispanic females [7]. The two most frequently used definitions for MetS are from the National Cholesterol Education Programs Adult Treatment Panel III (NCEP ATP III) and the International Diabetes Federation (IDF). According to the NCEP ATP III MetS is defined as the presence of three or more of any of the following criteria in an individual: high waist circumference (WC), elevated fasting glucose (GLU), low high-density lipoprotein cholesterol (HDL-C), elevated triglycerides (TG) and elevated blood pressure (BP) [3]. The International Diabetes Federation (IDF) requires central obesity as a mandatory component. The IDF defines MetS as high WC accompanied by any two of the four additional risk factors: elevated GLU, decreased HDL-C, elevated TG and elevated BP [11,12].

In the last decade the rates for obesity among the 18–29 year old population with some college education have increased significantly [13,14]. Further, there are ethnic disparities in the prevalence of obesity and obesity related risk for chronic diseases even in young adults [15]. Estimation of the rate of prevalence of MetS in young adults across all ethnicities indicates a range from 0.6-13% [16-21].

Young adults entering college are in a critical transition. It is well documented that first year college students experience weight gain faster than an average adult [22,23]. Various studies have indicated the poor health and lifestyle choices of college students, such as unhealthy diets, lack of physical activity and regular exercise, use of tobacco, and alcohol consumption. Many of these factors have been shown to contribute toward increasing the risks for MetS [24,25]. However, college students are an understudied population despite the reports of substantial risk for obesity and related chronic diseases [18,20]. A few studies focusing on young adults attending college at various locations in the US have reported prevalence rates for MetS ranging from 1.3% to 6.8% [17,18,21,23]. Most of these studies had predominantly Caucasian participants, [17,18,20,21,24,25]. Further, there are very few studies that have focused on the evaluation of risk for MetS in college students attending HBCUs which are mostly attended by AA young adults [26].

According to the 2010 census, more than 12 million young adults aged 18–24 were enrolled in colleges and universities in the United States [27]. About 16% of all AA higher education students in the nation are enrolled in the HBCUs, which comprise 3% of all colleges and universities nation-wide. Typically, most of the HBCUs have an enrollment of about 3,000 undergraduate students who are predominantly AAs [28]. The primary objective of the study was to evaluate the prevalence of MetS using the NCEP ATP III as well as the IDF definitions in a predominantly AA student population. The secondary objective was to evaluate which of the MetS criteria were most prevalent among this population, their distribution on the basis of the BMI status and assess the gender differences. The present study includes a comprehensive investigation of anthropometric, clinical and life style related aspects in first year students attending an HBCU.

## Methods

### Subjects

Undergraduate students aged 18–24 years were recruited through the ‘Student Health Awareness and Prevention Evaluation: an Undergraduate Program at Kentucky State University (SHAPE UP KSU), launched in the fall of 2009 to date. The design and the proposed protocols for the SHAPE UP KSU Program were approved by Kentucky State University’s Institutional Review Board. Each fall since 2009, freshmen students were recruited through various on campus avenues, primarily from Freshmen Orientation Program and various sections of the University Orientation Class (UNV101) and Health Fairs. Students were addressed in their classrooms regarding the features of the SHAPE UP KSU Program and invited to participate. Students were informed about the participation being voluntary, available to 18–24 years old and ineligible if pregnant or lactating. Willing students signed a written consent to participate. Participants were also informed that they could withdraw at any time during the course of the evaluations without any adverse consequences. Participant identifications were coded and records were maintained in a designated locked facility. In the three years of the study, 812 students offered their consent to participate, while 376 (92%AA) students consisting of 158 males (42%) and 218 females (58%) completed all the required assessments.

### Anthropometrics

All measurements were performed by one well trained registered nurse in a private setting and obtained following a 12 hour minimum fast, wearing light clothing without shoes and/or socks. Each measurement was conducted in duplicate unless variance between the measurements exceeded the standards, in which case, the measurement was repeated. The average of two readings was recorded. Height was recorded to the nearest 0.1 cm using a Seca Rod 220 stadiometer (Seca, Hamburg, Germany). Weight was recorded using a calibrated TANITA, (TANITA, Arlington Heights, Il.) scale to the nearest 0.1kg. BMI was calculated using the formula: weight in kilograms/height in meters^2^ (kg/m^2^). BMI classifications used were underweight (<18.5 kg/m^2^), normal (18.5-24.9 kg/m^2^), overweight (25–29.9 kg/m^2^), and obese (≥30.0 kg/m^2^). Subject’s waist circumference (WC) was measured at the top of the iliac crest upon exhalation to the nearest 0.1 cm using Gulick fiberglass, non-stretchable tape measure with a tensometer (average of three measures) (Patterson Medical, Mount Joy, PA). The North American WC cutoffs for all the participants were used.

### Biochemical and clinical assessments

All measurements were performed by one well trained registered nurse. After a 12 hour minimum fast, students reported to the pre-assigned, clinical setting in their respective residence hall for a pre-scheduled 15 minute screening appointment. On arrival, the participant was seated and offered a health risk survey/questionnaire. After a five minute resting phase, systolic and diastolic blood pressure was recorded using an automated monitor (HEM 757, Omron Health Care Inc, Bannockburn, IL). Blood pressure measurements were performed in sitting position, recorded in duplicate, 5 minutes apart. If variance between the two measurements exceeded the standard of 2 mmHg, the measurement was repeated. Mean values of the two readings were recorded. For biochemical tests, 12 hour fasting blood samples were assayed by standard methods for GLU, TC, HDL-C and TG, using an automated point-of-care desktop portable analyzer (Cholestech LDX, Hayward, CA). Using a combination of enzymatic methodology and solid phase technology, the analyzer measures a complete lipid and glucose profile in the blood sample within 5 minutes. The instrument was calibrated before every use. The method is certified by the Centers for Disease Control and Prevention’s (CDC) Cholesterol Reference Method Laboratory Network (CRMLN) and CDC’s Lipid Standardization Program (LSP) [29,30]. LDL-C was calculated by the Friedewald equation, excluding samples with triglyceride values > 400 mg/dL [31]. If the values of any of the tested parameters were found abnormal, another sample was drawn from the same participant within a week’s time. Mean values of the two readings were recorded. The participating students were given a copy of their results and if values were found outside the normal range, they were strongly encouraged to follow up with their health care provider.

### Metabolic syndrome criteria

The following NCEP ATP III criteria for MetS were used [3,4]. Increased abdominal fat WC (>102 cm for males and > 88 cm for females), elevated TG (≥ 150 mg/dL), low HDL-C (< 40 mg/dL for males and <50 mg/dL for females), elevated fasting GLU (≥ 100 mg/dL), and systolic arterial BP ≥ 130 mmHg and/or diastolic arterial BP ≥ 85 mmHg. Individuals with 3 or more of the criteria were classified with MetS. The following IDF definition for MetS was used. Individuals with increased abdominal fat WC (>102 cm for males and > 88 cm for females), and any of two or more of the following criteria such as elevated TG (≥ 150 mg/dL), low HDL-C (< 40 mg/dL for males and <50 mg/dL for females), elevated fasting GLU (≥ 100 mg/dL), and systolic arterial BP ≥ 130 mmHg and/or diastolic arterial BP ≥ 85 mmHg were classified with MetS.

### Statistical analysis

SPSS software version 19.0 (SPSS, Chicago, IL) was used for analysis. Demographics were calculated using means and frequencies. Analysis of variance (ANOVA) scores on the Means procedure were used to examine differences between genders for all anthropometric, clinical and biochemical parameters. The cross tab procedure and Pearson’s chi-square tests and non-parametric independent t-test were used to analyze the prevalence of MetS criteria per gender, the number of MetS criteria per BMI and prevalence of MetS criteria per BMI category. Significance was set at *p ≤* 0.05 for all tests.

## Results

**Figure 1:**
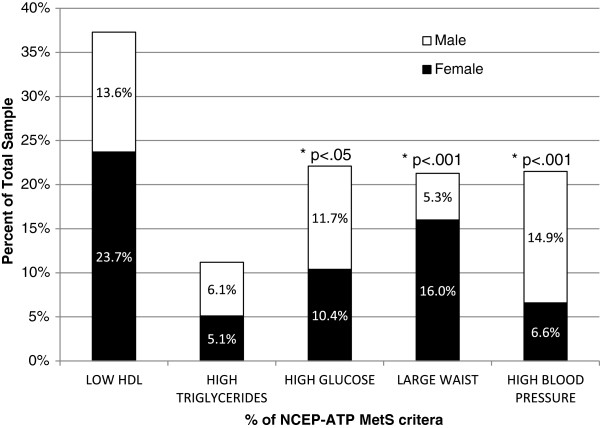
**Percentage of NCEP-ATP MetS criteria by gender.** Data analyzed using Crosstabs Procedure, Pearson Chi-Square test for significance, non-Parametric Independent t-test. Female n=218; Male n=158. *p ≤ 0.05.

Participant demographics and individual MetS criteria along with the differences in the anthropometric, clinical and biochemical parameters between males and females participating in the study are shown in Table 1. The majority of the participating subjects were AA (91.2%), followed by Caucasians (5.6%), and others (3.2%), with mean age of 19.88 years. Approximately 58% were females (n=218) and 42% were males (n=158). More than 85% of the participants resided in the residence halls that were not equipped with in room kitchen facilities, while the remaining resided in private housing. Although approximately 46.2% (n=174) of the participants were in the normal BMI range, 26.3%, (n=99) were in the overweight range, while 25% (n=94) were obese. The remaining 2.5% (n=9) were underweight. The Mean BMI was in the overweight range (26.94±6.9 kg/m^2^). Figure 1 shows the most prevalent MetS parameters in the total sample, which were low HDL-C (37.3%), high GLU (22.1%), high BP, large WC and high TG. It is interesting to note that despite the higher prevalence, no statistical difference was observed between males and females for HDL-C values. However, statistical significance was observed between the genders for the distribution of other three parameters. More males than females had elevated fasting GLU and elevated blood pressure, while more females had high WC than males.

**Table 1:** Anthropometric, clinical, and biochemical description of the subjects

**Characteristics**	**Total (*n *= 376)**	**Female (*n *= 218)**	**Male (*n *= 158)**
	**Mean ± *SD***	**Mean ± *SD***	**Mean ± *SD***
Age (years)	19.9±3.6	19.9±3.6	19.9±3.6
BMI (kg/m^2^)	26.9±6.9	27.8±7.5	25.7±5.8**
WC (cm)	81.5±15.5	81.7±16.2	81.2±14.4
SBP (mm Hg)	120.8±14.3	115.5±11.7	128.1±14.3**
DBP (mm Hg)	74.1±10.2	73.6±9.7	74.8±10.9
TC (mg/dL)	154.0±30.7	156.0±31.6	151.3±29.4
LDL-C (mg/dL)	89.2±28.0	89.6±27.8	88.7±28.2
HDL-C (mg/dL)	50.0±14.3	52.8±14.2	46.1±13.5**
TG (mg/dL)	87.3±47.4	81.6±45.0	95.1±49.7**
GLU (mg/dL)	93.4±12.6	91.8±11.3	95.6±13.8**

SD = Standard Deviation; BMI = body mass index; WC = waist circumference; SBP = systolic blood pressure; DBP = diastolic blood pressure; TC = total cholesterol; LDL-C = low-density lipoprotein cholesterol; HDL-C = high-density lipoprotein cholesterol; TG = triglycerides; GLU = glucose.

Data analyzed using ANOVA tests to determine gender differences.

**p < 0.01 indicates differences between males and females.

**Figure 2:**
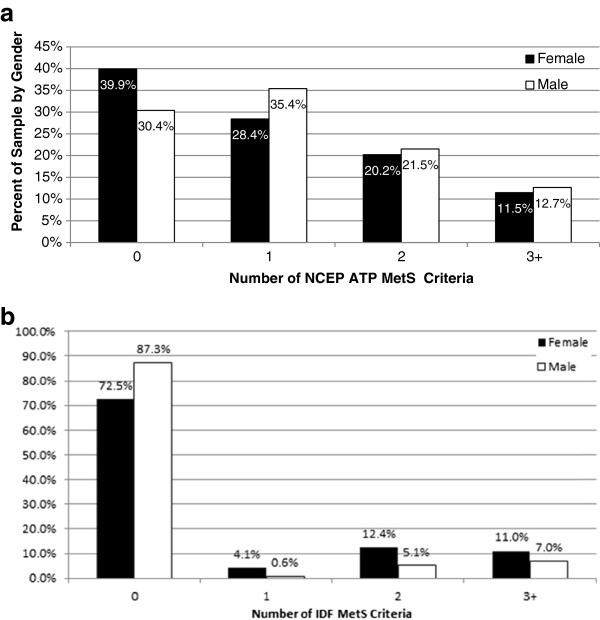
**(a) Prevalence of NCEP ATP MetS criteria by gender.** Data analyzed using Crosstabs Procedure, Pearson Chi-Square test for significance, non-Parametric Independent t-test. Female n=218; Male n=158. **(b)** Prevalence of IDF MetS criteria by gender. Data analyzed using Crosstabs Procedure, Pearson Chi-Square test for significance, non-Parametric Independent t-test. Female n=218; Male n=158.

Based on NCEP ATP III definition, the prevalence of MetS in the total sample was 12%, with 11.5% of females and 12.7% males having MetS (Figure 2a). A total of 31.4% ( n=118) of the sample had at least one metabolic dysfunction and 20.7% (n=75) of the sample had at least two metabolic dysfunctions. Approximately 3% of the total population (n=11) had four MetS criteria present, while 1% (n=4) had all five criteria present.

Based on IDF definition, the prevalence of MetS in the total sample was 9.3% with 11% females and 7% males having MetS (Figure 2b). A total of 21.3% of the sample (n=80) had the definitive metabolic dysfunction (large WC) and 9.3% of the total sample had two metabolic dysfunctions (n=35).

**Figure 3:**
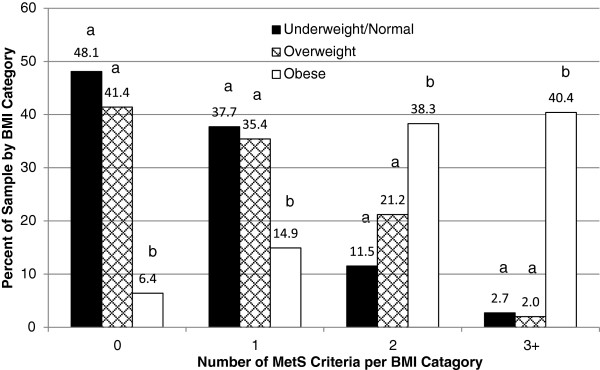
**Number of MetS criteria per BMI category.** Data analyzed using Crosstabs Procedure, Pearson Chi-Square test for overall significance, ONEWAY POSTHOC=TUKEY SCHEFFE LSD (p≤0.05) (independently) Non-Parametric Independent t-test (independently). a= Difference in the values of the variables not statistically different, b=Difference in the values of the variables are statistically significant.

**Figure 4:**
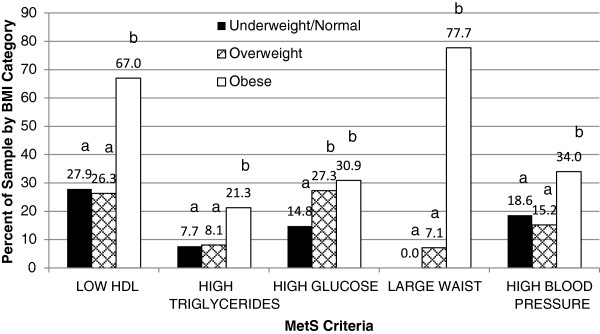
**Prevalence of MetS criteria per BMI category.** Individual MetS Criteria by BMI Status were analyzed using Crosstabs Procedure, Pearson Chi-Square test for overall significance ONEWAY POSTHOC=TUKEY SCHEFFE LSD (p≤0.05) (independently). Non-Parametric Independent t-test independently). a= Difference in the values of the variables not statistically different, b=Difference in the values of the variables are statistically significant.

Individual’s BMI status played a significant role in predicting the risk for MetS, as 48% of the subjects with normal BMI (<25 kg/m^2^) had no criteria for MetS, and 41.4% overweight subjects (BMI > 25–30 kg/m^2^) had no criteria for MetS. However, only 6.4% obese subjects (BMI≥ of 30 kg/m^2^) indicated no criteria for MetS. Approximately 40% of obese subjects had three or more criteria for MetS, a much higher prevalence than found among subjects in the normal or overweight categories (2.7% and 2% respectively) (Figure 3). As expected, the obese group had the highest prevalence of elevated WC (77%), low fasting HDL-C (67%), elevated blood pressure (34.5%) and elevated fasting GLU (30.9%) compared to those in underweight, normal and overweight categories (Figure 4). However, not limited to the obese group, a statistically higher risk for elevated fasting GLU was observed even in the overweight subjects.

## Discussion

According to the CDC’s 2011Youth Risk Behavior Surveillance data on adolescent high school students, AA females were found to be 1.4 times more likely to be overweight and 2.4 times more likely to be obese than Caucasian females. Similarly, AA males were 1.6 times more likely to be obese than Caucasian males [32]. However, there are relatively few similar studies in college students in general, especially AA college students. This is one of the first comprehensive studies with the largest sample size among any studies evaluating the prevalence of MetS among college students and reporting the prevalence of metabolic risks integrating anthropometric, clinical and biochemical parameters in a predominantly AA student population attending an HBCU.

Using the NCEP ATP III criteria, the overall prevalence of MetS in the sample was 12%. When the IDF definition was applied, the overall prevalence of MetS in the sample was 9.3%. Irrespective of the definition used, the overall prevalence of MetS in this sample population located in the South/South-east is significantly higher than any other college based study.

Unlike other reports on college student population, majority (51.3%) of the sample population in the current study was overweight (26.3%) with more females than males being overweight and/or obese (25%) [17,18,20,21,23-25]. The most prevalent criterion in this sample was low HDL-C (37.3%), which is quite common in college students. Dalleck and Kjelland have reported an occurrence rate of 47.3% for low HDL-C in their study sample [21]. Though the reference values for HDL-C being different for males and females, the difference in the MetS prevalence between the genders was not as large as found in the NHANES 2003–2006 report [7]. To the best of our knowledge, again, this is also the first study to report the highest occurrence rate (22%) for pre-diabetes (elevated GLU) among studies on college students [17,18,20,21,23,24]. The prevalence of individuals with one (NCEP 31.4% and IDF-21.3%) and two (NCEP 20.7% and IDF 8.75%) MetS components in the present study are considerably higher than previously reported. Using NCEP criteria, Huang *et al.* have found lower occurrences of MetS risk factors in a primarily white college student population, with 25.2% and 1.2% possessing 1 and 2 MetS components respectively, while Fernandes and Lofgren reported a prevalence of individuals with either one or two of the MetS criteria to be 28% and 7.7% respectively in their sample of college students in the North-east [18,25]. Dalleck and Kjelland reported a prevalence of individuals with either one or two of the MetS criteria of 47.2% and 13.0% respectively, from a college in the Midwest region of the US which was the highest so far [21]. Further, significant differences on the basis of BMI were found in the sample, with those in the obese category reporting overall worse outcomes. The current study offers the evidence of ethnic differences in the distribution of individual risk factors in the overall prevalence of MetS and its individual criteria in college student populations along ethnic and geographical lines. The trends typified the distribution of obesity and diabetes as these health conditions, which are more concentrated in the South and Southeast of the US and severely impact AAs, including young adults [7].

Though the national data from NHANES (2003–2006) reported elevated WC and elevated BP as the most prevalent risk factors in population in this age group, most studies in young adults have reported greater occurrence of low HDL-C and elevated TG [17,18,20,21,23,24]. Further it is important to note that the same NHANES study also demonstrated racial differences in the WC cut points that correspond to increased risk for Mets (BMI of 25 kg/m^2^). In AAs the WC corresponding to a BMI of 25 kg/m^2^ were 86.4 cm in men and 83.5 cm in women compared with 91.3 cm and 83.4 cm in Caucasian American men and women respectively [33]. The IDF attempts to define MetS in younger populations worldwide [34]. When we applied these cut off values for WC to AA and Caucasian participants in our sample population, more overweight and obese individuals were identified than previously (approximately 14.5% overweight and 19% obese). On applying the IDF definition, incorporating these racial differential values for WC, the total prevalence of MetS in the student population rose to 11.7% from 9.3%. Thus, it is important to identify the racial differences in the WC that confer additional risk in young adults in this age group. In order to better identify those with risk for MetS, our data suggest the need for setting age appropriate cut off points in young AA populations rather than those that are currently in use.

Overall, our study corroborates the evidence of occurrence of metabolic precursors of CHD in this young a population. It is disturbing to observe such high rates of pre-diabetes in very young adults which represents a serious public health concern. According to the American Heart Association, adults with diabetes alone are two to four times more likely to have heart disease or a stroke [35].

Although college years lay the foundation for future health behaviors and health status, and are the years in life when health education and preventive care may arguably have their greatest impact, typically college aged students perceive themselves to be ‘invincible’. Further, they do not always fully comprehend the seriousness of the prevailing risk factors such as diabetes and its associated risks for debilitating quality of life and chronic health conditions. It has been estimated that the average annual medical cost of treating an adult with risk factors for CHD is approximately $5500 annually [36]. The findings of the current study reveal the need for additional screening of college students, especially those with family history of CHD that can provide more information so as to better design well targeted interventions to decrease the risk of MetS and eventual, yet an early onset of CHD. The data from the current and other studies indicate that it is necessary to design prevention and early intervention programs primarily targeting risk factors such as low HDL, elevated TG and high WC, and also focus on better pre-diabetes and diabetes self-management. MetS being a cluster of criteria, there is a significant likelihood that additional criteria will develop later in life if they are not addressed in time with due modifications in lifestyles [4,17,18].

There are several limitations to this study. Some inherent selection bias among the participant was unavoidable due to the following factors; Student motivation/participation rate: Those who volunteered carried a positive perception of body image, while those who knew they were either overweight or obese, opted not to participate. Retention Rate: On an average, almost 50% of the freshmen class would sign up to participate every year, while less than half of those completed all the screening procedures. A few identified reasons behind this phenomenon are: 1. Participation being voluntary and not mandatory, 2. Poor retention rates at the school level (44%), 3. Not all instructors of the University Orientation Class (UNV101) offered extra points for students’ participation, and 4. As mentioned earlier, those who knew they were either overweight or obese opted not to participate.

## Conclusions

The prevalence of MetS among this predominantly AA college student population is significantly high and the undiagnosed rates can be alarming. Lifestyle choices and modifications such as increased physical activity and dietary changes can improve HDL-C, TG and GLU concentrations in this population. However, currently there is no practice of screening college students for chronic diseases. In order to fill the current void, there is a critical need for colleges in general, especially HBCUs and other minority serving institutions, to implement health screenings and educational initiatives. Colleges that have dropped the pre-admission physical examination requirement may want to re-establish screening programs to identify at risk students at the beginning of the academic years. Such screening programs especially across the HBCU system will help in designing of more effective prevention and early intervention strategies with a focus on achieving health goals.

## Abbreviations

MetS: Metabolic Syndrome; HBCU: Historically Black Colleges and Universities; AA: African American; NCEP- ATP III: National Cholesterol Education Program’s Adult Treatment Panel III; CHD: Coronary Heart Disease; US: United States; NHANES: National Health Awareness and Nutrition Education Survey; BMI: Body Mass Index; WC: Waist Circumference; GLU: Glucose; HDL-C: High Density Lipoprotein- Cholesterol; TG: Triglyceride; BP: Blood Pressure; IDF: International Diabetes Federation; SHAPE UP KSU: Student Health Awareness and Prevention Evaluation: an Undergraduate Program at Kentucky State University; UNV: University Orientation; CDC: Centers for Disease Control and Prevention; CRMLN: Cholesterol Reference Method Laboratory Network; LSP: Lipid Standardization Program; ANOVA: Analysis of Variance.

## Competing interests

There are no competing interests.

## Authors’ contributions

AT made a substantial contribution to the conception and study design. AT and PR were involved in data collection. AT was involved in refining the study design, statistical analysis and drafting the manuscript. PR critically revised the manuscript. All authors read and approved the final manuscript.

## References

[B1] Reaven GM Role of insulin resistance in human disease Diabetes 1988 37 1595 1607 10.2337/diabetes.37.12.1595 3056758

[B2] Grundy SM Brewer HB Jr Cleeman JI Smith SC Jr Lenfant C Definition of metabolic syndrome: report of the National Heart, Lung, and Blood Institute/American Heart Association conference on scientific issues related to definition Circulation 2004 109 433 438 10.1161/01.CIR.0000111245.75752.C6 14744958

[B3] Executive summary of the third report of the National Cholesterol Education Program (NCEP) Expert Panel on Detection Evaluation, and Treatment of High Blood Cholesterol in Adults (Adult Treatment Panel III) JAMA 2001 285 2486 2497 10.1001/jama.285.19.2486 11368702

[B4] Grundy SM Cleeman JI Daniels SR Donato KA Eckel RH Franklin BA Gordon DJ Krauss RM Savage PJ Smith J Spertus A Costa F Diagnosis and management of the metabolic syndrome: an American Heart Association/National Heart, Lung, and Blood Institute Scientific Statement Circulation 2005 112 2735 2752 10.1161/CIRCULATIONAHA.105.169404 16157765

[B5] Reaven GM The metabolic syndrome: is this diagnosis necessary? Am J Clin Nutr 2006 83 1237 1247 16762930 10.1093/ajcn/83.6.1237

[B6] Third Report of the National Cholesterol Education Program (NCEP) Expert Panel on Detection Evaluation, and Treatment of High Blood Cholesterol in Adults (Adult Treatment Panel III), final report Circulation 2002 106 3143 3421 12485966

[B7] Ervin RB Prevalence of Metabolic Syndrome Among Adults 20 Years of Age and Over, by Sex, Age, Race and Ethnicity, and Body Mass Index: United States, 2003–2006 Nat Hlth Stat Rep 2009 13 1 7 19634296

[B8] Crist LA Champagne CM Corsino L Lien LF Zhang G Young DR Influence of change in aerobic fitness and weight on prevalence of metabolic syndrome Prev Chronic Dis 2012 9 110 171 10.5888/pcd9.110171 PMC3368700 22405475

[B9] Minino AM Heron MP Murphy SL Kochanek KD Deaths: final data for 2004 Natl Vital Stat Rep 2007 55 1 119 17867520

[B10] Heron M Deaths: Leading Causes for 2008 Natl Vital Stat Rep 2012 60 1 94 22827019

[B11] Ford ES Giles WH Dietz WH Prevalence of the metabolic syndrome among US adults: findings from the third National Health and Nutrition Examination Survey JAMA 2002 287 356 359 10.1001/jama.287.3.356 11790215

[B12] Program NCE Executive summary of the third report of the NCEP expert panel on detection, evaluation, and treatment of high blood cholesterol in adults (Adult Treat Panel III) JAMA 2002 285 2486 2497 10.1001/jama.285.19.2486 11368702

[B13] Mokdad AH Serdula MK Dietz WH Bowman BA Marks JS Koplan JP The spread of obesity epidemic in the United States, 1991–1998 JAMA 1999 282 1519 1522 10.1001/jama.282.16.1519 10546690

[B14] Mokdad AH Ford ES Bowman BA Dietz WH Vinicor F Bales VS Marks JS Prevalence of obesity, diabetes and obesity related health risk factors, 2001 JAMA 2003 289 76 79 10.1001/jama.289.1.76 12503980

[B15] Wang Y Beydoun MA The Obesity Epidemic in the United States—Gender, Age, Socioeconomic, Racial/Ethnic, and Geographic Characteristics: A Systematic Review and Meta-Regression Analysis Epidemiol Rev 2007 29 1 6 28 10.1093/epirev/mxm007 17510091

[B16] Mattsson N Ronnemaa T Juonala M Viikari JS Raitakari OT The prevalence of the metabolic syndrome in young adults. The Cardiovascular Risk in Young Finns Study J Intern Med 2007 261 159 169 17241181 10.1111/j.1365-2796.2006.01752.x

[B17] Huang TT Kempf AM Strother ML Li C Lee RE Harris KJ Kaur H Overweight and components of the metabolic syndrome in college students Diabetes Care 2004 27 3000 3001 10.2337/diacare.27.12.3000 15562226

[B18] Huang TT Shimel A Lee RE Delancey W Strother ML Metabolic risks among college students: prevalence and gender differences Metab Synd Relat Disord 2007 5 365 372 10.1089/met.2007.0021 18370807

[B19] Yen SL Chiu TY Lin YC Lee YC Lee LT Huang KC Obesity and hepatitis B infection are associated with increased risk of metabolic syndrome in university freshmen Int J Obes (Lon) 2008 32 474 480 10.1038/sj.ijo.0803753 17955029

[B20] Burke JD Reilly RA Morrell JS Lofgren IE The University of New Hampshire’s Young Adult Health Risk Screening Initiative J Am Diet Assoc 2009 109 1751 1758 10.1016/j.jada.2009.07.005 19782175

[B21] Dalleck LC Kjelland EM The prevalence of Metabolic Syndrome and Metabolic Syndrome risk factors in college-aged students Am J Hlth Prom 2012 27 1 37 42 10.4278/ajhp.100415-QUAN-116 22950924

[B22] Holm-Denoma JM Joiner TE Vohs KD Hearthertob TF The freshman fifteen (the freshman five actually): predictors and possible explanations Health Psychol 2008 27 S3 S9 18248103 10.1037/0278-6133.27.1.S3

[B23] Levitsky DA Halbmaier CA Mrdjenovic G The freshman weight gain: a model for the study of the epidemic of obesity Int J Obes Relat Metabol Disord 2004 28 1435 1442 10.1038/sj.ijo.0802776 15365585

[B24] Spencer L Results of a heart disease risk-factor screening among traditional college students J Am Coll Hlth 2002 50 6 291 296 10.1080/07448480209603447 12701654

[B25] Fernandes J Lofgren IE Prevalence of Metabolic Syndrome and individual criteria in college students J Am Coll Hlth 2011 59 4 313 321 10.1080/07448481.2010.508084 21308592

[B26] Kelly GA Lowning L Cardiovascular disease risk factors in Black college students J Am Coll Health 1997 45 4 165 170 10.1080/07448481.1997.9936877 9019003

[B27] U.S. Department of Commerce United States Census Bureau. CPS October 2010. Detailed tables 2010 http://www.census.gov/hhes/school/data/cps/2010/tables.html

[B28] U.S. Department of the Interior What are Historically Black Colleges and Universitieshttp://www.doi.gov/hrm/black.html

[B29] Shepherd MD Mazzachi BC Shephard AK Comparative performance of two point-of-care analysers for lipid testing Clin Lab 2007 53 9–12 561 566 18257461

[B30] Carey M Markham C Gaffney P Boran C Maher V Validation of a point of care lipid analyser using a hospital based reference laboratory Ir J Med Sci 2006 175 4 30 35 10.1007/BF03167964 17312826

[B31] Friedwald WT Levy RI Fredrickson DS Estimation of the concentration of low density lipoprotein cholesterol in plasma, without use of the preparative ultracentrifuge Clin Chem 1972 18 499 502 4337382

[B32] US Department of Health and Services Obesity and African Americans http://minorityhealth.hhs.gov/templates/content.aspx?ID=6456

[B33] Zhu S Heymsfield SB Toyoshima H Wang Z Pietrobelli A Heshka S Race/ ethnicity- specific waist circumference cutoffs for identifying cardiovascular disease risk factors Am J Clin Nutr 2005 81 409 415 15699228 10.1093/ajcn.81.2.409

[B34] The IDF consensus definition of the Metabolic Syndrome in children and adolescents 2007 http://www.idf.org/webdata/docs/Mets_definition_children.pdf

[B35] The American Heart Association Cardiovascular Disease and Diabetes http://www.heart.org/HEARTORG/Conditions/Diabetes/WhyDiabetesMatters/ Cardiovascular-Disease-Diabetes_UCM_313865_Article.jsp

[B36] Sullivan PW Ghushchyan V Wyatt HR Hill JO The medical cost of cardiometabolic risk factor clusters in the United States Obesity 2007 15 3150 3158 10.1038/oby.2007.375 18198326

